# Dr. Gerhard on Diseases of the Heart and Aorta—with Cases

**Published:** 1842-04-23

**Authors:** W. W. Gerhard


### MEDICAL EXAMINER.

NEW SERIES.

No. 17.] PHILADELPHIA, APRIL 23, 1842. [Vol. I.

### *Diseases of the Heart and Aorta.* By W. W. Gerhard, M. D.

(Cases reported by T. L. Walker, M.D.)

*Case 1.—Aneurism of Aorta—Dilatation and Hypertrophy of Heart—Tuberculous Disease of Lungs, following an acute attack—Diagnostic Signs of Aneurism of the Arch.*

R. R., coloured man, æt. 43, admitted on the 16th of Nov. 1841. Had inguinal hernia twelve years, otherwise perfectly healthy till last March. After exposure, cough, hoarseness, and a wandering pain of chest, chiefly confined to upper mediastinum, seized him. During past summer constipation and palpitations, with a sense of suffocation at times. As first seen, general emaciation; countenance anxious; breathing difficult; nostrils dilated; cough frequent, expectorates frothy mucus; voice harsh; tongue slightly coated. Pulse regular, small and feeble, 108.

*Physical Signs.*—Some fulness of left side. Percussion dull throughout, flat at bottom, nearly flat upon upper part of sternum; respiration feeble throughout left lung; puerile through right, with some mucous rhonchus. First sound of heart blowing and prolonged; second indistinct.

*Pres.* Pulv. Digitalis Hyd. C. Mit. et Ipecac.

Dec. 12th.—Slight ptyalism; pulse slower, 96, fuller; percussion on left side more resonant; still very dull over upper part of sternum; sounds of heart nearly natural. General improvement; no medicine; generous diet.

27th.—Strength gradually declined; emaciation increased; cough worse; expectorates nummular matter; percussion clear on left side, except over region of heart and sternum, from its top to near the middle, where it is flat; flatness extends one and a half inches beyond median line. Respiration at summit of right lung cavernous, with gurgling. Faint bruit de scie at the upper part of the sternum, corresponding with the flatness.

Feb. 7th, 1842.—Since 27th December strength and flesh wasted rapidly; cough continued troublesome, with abundant nummular expectoration; countenance very dejected; almost perfect aphonia, with deafness; orthopnœa; appetite gone; tongue fissured; pulse small and feeble, 120; extremities cold; cavernous respiration at summit of right lung, with crackling throughout upper lobe. Remained in the above distressed state till evening of the 9th, when he died.

*Autopsy twenty-four hours after death.*—Large limpid serous effusion in left pleura—a similar effusion in pericardium; left pleura adherent throughout by firm and semicartilaginous adhesions; a mass investing the trachea and top of sternum, filling upper portion of chest, was next observed. It was found to be a tumour five and a quarter inches in breadth, by four and five-eighth inches in length. When the aorta was opened the scissors passed into the tumour, which was a large aneurismal sac with the usual coagulum. The dilatation began two inches beyond aortic valves, and the aneurism terminated at the sixth dorsal vertebra. At upper portion of sac, the coagulumfor the thickness of one and three-eighths of an inch, consisted of white fibrous laminæ, the remaining half inch of softer reddish lymph ; cavity would contain a body about the size of fœtal head. Below, the artery is of a dark reddish tint, thickened with yellow patches of cartilaginous matter. Heart dilated throughout; right ventricle filled with soft coagulum ; internal membrane slightly reddened, firm ; membranes of valves of a normal tint; left ventricle filled with dark coagulum ; tissue soft ; mitral valve slightly thickened with some old deposits, but no recent vegetations; semilunar valves reddened, soft, not thickened.

At summit of right lung a large cavity with double membrane ; several smaller cavities communicate in rest of upper lobe, containing red sanious matter ; numerous tubercles in two lower thirds of right lung ; in anterior portion tubercular infiltration almost displacing parenchyma; lung looks as if it was injected with wax. In stomach marks of chronic inflammation, particularly at pyloric orifice. Colon contracted through its whole extent. Liver paler than usual. Spleen and kidneys perfectly normal.

This case was of extreme interest from the difficulty in the diagnosis at the time of the entrance of the patient. The cough was then very violent, and the dyspnœa occurred in irregular paroxysms. As the disease advanced the cough increased, with yellow puriform sputa, thick and paste-like, as in most cases of rapid tuberculous softening. This expectoration coincided with crackling, and finally with gurgling at the summit of the right lung. At his entrance the respiration was blowing at the summit of the lung, and the percussion dull; there was therefore evidently a tuberculous deposition, which had softened while the patient remained in the hospital. But there were other symptoms which could not be accounted for by the tuberculous disease. The palpitation of the heart was at times considerable, but not constant; the pulse offered no aneurismal thrill ; the impulse of the heart was increased, but not to an extreme degree; the sounds were both altered. It was evident, therefore, that the patient laboured under hypertrophy and dilatation. The second sound was much less affected than the first, because the semilunar valves were comparatively little altered. There remained the dull sound on percussion at the upper portion of the sternum, and the peculiar harshness of the voice, with paroxysms of coughing, and on one occasion a feeble though distinct bellows sound. The dulness on percussion could be explained only by the presence of a tumour beneath the sternum. The harsh peculiar tone of the voice, particularly after speaking for a short time, when the inspirations became long and noisy, almost as in whooping cough, and the paroxysmal nature of the cough could be explained also, only on the supposition that a tumour was pressing upon the trachea : the same alteration of the voice and paroxysmal cough occur in cases of enlarged bronchial glands, when their size is considerable enough to compress the trachea; and if the symptoms had occurred in a child, it would have been natural to refer them to this cause; but as such enlargements of the glands are rare in adults, it was much more probable to suppose that the tumour was aneurismal. This diagnosis was given at an early period to some of the clinical class; (by one of them, Dr. Foree of Kentucky, these notes were preserved;) after the development of the bruit de scie the diagnosis was rendered more certain, but not altered.

The signs of aneurism of the aorta are often obscure. The strong pulsation which is often felt, depends merely upon the direction of the current of the blood; in the present case the form and situation of the fibrinous coagulum was such as to prevent the thrill.

The most important symptoms were therefore the dull percussion and the peculiar tone of the voice, resulting from the pressure upon the trachea by the aneurismal tumour. I had previously observed the same symptom in another case of aneurism, in which the symptoms were equally obscure; there was also the same paroxysmal asthmatic cough, very similar in both cases, and dependent upon the same cause—pressure in the trachea. The comparison of these different signs rendered the diagnosis nearly as certain as it is in those cases in which the tumour points externally, or the aneurismal thrill is extremely characteristic.

The origin of the case was remarkable. Making all due allowance for the possibility of previous aortic diseases, it is very certain that the symptoms either began, or at least suddenly increased after an acute attack of inflammation. This was undoubtedly aortitis, probably conjoined with endocarditis. The later discoveries have shown very conclusively that the larger number of heart diseases, especially those connected with valvular lesions, are the direct result of inflammation. The same rule holds good as to the majority of cases of thickening and dilatation of the aorta. The internal coat giving way, or the whole tissues of the artery becoming softened and losing their elastic resistance to the impulsion of the blood.

The therapeutics in a case so utterly hopeless, were of course almost nugatory. There is, however, a retrospective therapeutics; when the proper time of the application of remedies is lost, we may still deduce the rules necessary for their administration. When the symptoms of aortitis, such as pain, and strong pulsation and sawing sound, are found at the region of the aorta, we must continue the use of anti-phlogistic measures, and watch over the health of the patient until his entire restoration. Just as in cases of pleurisy with abundant effusion, the possibility of phthisis should be borne in mind, and the surest way of preventing it will be to watch the patient until the complete return of health.

*Case* 2.—*Pericarditis—Endocarditis—Acute Tuberculous disease of the Lungs.*

J. M., æt. 20, admitted Nov. 19th, 1842, Irishman; in America four months; well during passage; no palpitation; no shortness of breath; four weeks past got his feet wet and took cold; hoarseness two days after, then a cough, which has been dry since; lately expectorates a little mucus; no pain, except after coughing, then severe; can lie on either side.

As first seen, of moderate muscular development, slight febrile flush; dilatation of nostrils; respiration thirty-two, high and laboured; pulse 112, thrilling, regular and compressible; little motion of left side of chest; fulness at inferior portion of left side, none at precordial region; thrilling distinct in large arteries of neck; distension of veins of neck. Percussion, anteriorly on right, clear; on left, dull at summit, flat at fourth rib from sternum to an inch beyond left nipple; dull in axilla; purring felt over heart; second sound of heart almost lost; first sound, opposite semilunar valves, sharp and rough; over middle of heart more dulness with friction sound. Posteriorly of left side, respiration inferiorly feeble; no œgophony; percussion dull. ℞. Pulv. Digitalis, gr. ss. 3 in die. Cut cups over heart.

22.—No change of countenance, except less dyspnœa; no œdema; less thrill of heart; first sound prolonged bellows; the same heard in second sound; variable friction heard over sternum. ℞. Digitalis gr. $\frac{1}{2}$, 5 in die.

24.—Less oppression; sounds of heart as before. Treatment continued.

Dec. 3.—Improvement continues; no general symptoms, except cough; pulse still thrilling; bellows sound of heart diminished; impulse still diffused; flatness over heart perfect. ℞. Digitalis gr. ss.; Assafœt. gr. v. ter in die.

10.—With symptoms of pneumonia; cheeks flushed; no dilatation of nostrils; tongue moist, slightly coated; pulse more frequent and thrilling; cough great at night; bronchial respiration at lower posterior part of right lung, with subcrepitant rhonchus.

18.—Loose subcrepitant rhonchus below both clavicles; increase of dyspnœa, and flush of face; expectoration purulent and nummular; tuberculous action of heart blowing, but with less bellows sound.

20.—Face during day frequently flushed; cough oppressive; no chills; expectoration yellow, homogeneous and paste-like; some tremors; great fever; skin, except at flush, pallid; tongue moist, slightly sallow; bowels open. Percussion, anteriorly of left, dull; flatness over region of heart; percussion of right moderately clear; slightly dull at summit; mucous rhonchus heard at summit of left; inferiorly, traces of bronchial and mucus with vesicular; diffused action of heart over large space; loose crackling, with imperfect cavernous respiration at summit of right lung; inferiorly vesicular, with some mucous rhonchus; posteriorly, percussion dull at summit of both lungs; at summit of right, pectoriloquy and cavernous respiration heard; at left, crackling.

Jan. 10.—Since last date very little change; face flushed; pulse quick and frequent, 150; dyspnœa constant; no sweats or chills for a week; bowels open twice only during twenty-four hours; severe diarrhœa for some days; voice hoarse; tongue coated and moist; cough frequent, chiefly at night; expectoration purulent, gum-like and nummular; respiration high, 28; loose mucous crackling heard in both lungs. These symptoms were more or less severe until the 18th, when he expired.

*Autopsy twelve hours after death.*—Three pints of a thick ropy liquid in right pleura; right lung, externally, of a light gray colour; pleura thickened and firm, but contains no tubercles; tubercular infiltration in upper and middle lobes of the lung; much softened in the centre, where there are commencing cavities containing a white cheesy matter, exuding on pressure; lower lobe congested, bright red colour, infiltrated with tubercles, but less softened; left lung of bright red; injection throughout with abundant tubercular deposition; one large cavity at summit of lung; many smaller ones scattered through, making it cerebriform. Upwards of a pint of fluid in pericardium; opaque depositions of coagulable lymph on its surface, with a bright injected spot at upper part; heart little larger than natural; right ventricle natural, but contains a large organized coagulum; valves natural; left ventricle hypertrophied, and slightly dilated; its internal surface presents patches of redness, in most places of a pale dull tint; brighter near the valves than elsewhere. From the shading of the colour and the paleness of it in certain parts, the redness is evidently less than it must have been during the active stages of the inflammation. The mitral valve is thickened from deposition of organized pale lymph, intimately adhering to the internal membrane. The semilunar valves are of a much more intense red than the rest of the endocardium, with minute granulations of lymph closely united to them, and at the same time are thickened from interstitial deposition between their folds.

The mucous membrane of the stomach was slightly injected and softened; there was some patches of inflammatory redness in the upper portion of the small intestine.

The mucous membrane of the colon was of a pale slate colour, softened with superficial ulcerations. Cyrrhosis and hypertrophy of the liver; softening and enlargement of the spleen; kidneys lobulated, but essentially healthy.

From this sketch of the history and of the pathological appearances, it is plain that the case presents several points of resemblance with the last; the patient entered labouring under severe endo-pericarditis, which gradually subsided without entirely disappearing. During the acute period of the disease the attention of the clinical class was frequently called to the case, as a remarkable illustration of the symptoms of acute cardiac inflammation. There were, however, some unusual symptoms; the pulse was extremely frequent, and offered the peculiar quick jerking movement so common in acute phthisis; the flush of the cheek was more constant and brighter than in ordinary cardiac inflammation, and the sweats were more frequent. As the phthisis advanced these symptoms became more decided, and those which were immediately connected with the heart, diminished in intensity.

There is no doubt that the tuberculous disease of the lungs was already forming at the time of the patient's entrance; probably its origin was simultaneous with inflammation of the heart; that is, the acute tuberculation was accompanied with a local inflammation of the serous membranes of the chest; but instead of its being limited to the pleuræ, the membranes of the heart were the seat of it. The early development of the tuberculous disease explains the inefficacy of the treatment; for although the uncomplicated serous inflammations are in general readily removed, those cases in which they are connected with acute tubercles are most intractable.

